# Self-Harm, Suicidal Ideation, and Suicide Attempts in Chinese Adolescents Involved in Different Sub-types of Bullying: A Cross-Sectional Study

**DOI:** 10.3389/fpsyt.2020.565364

**Published:** 2020-12-03

**Authors:** Chang Peng, Wenzhu Hu, Shanshan Yuan, Jingjing Xiang, Chun Kang, Mengni Wang, Fajuan Rong, Yunxiang Huang, Yizhen Yu

**Affiliations:** ^1^Department of Maternal, Child and Adolescent Health, School of Public Health, Tongji Medical College, Huazhong University of Science and Technology, Wuhan, China; ^2^First Clinical School, Tongji Medical College, Huazhong University of Science and Technology, Wuhan, China; ^3^Department of Social Medicine and Health Management, Xiangya School of Public Health, Central South University, Changsha, China

**Keywords:** adolescents, suicide attempts, suicidal ideation, self-harm, bullying

## Abstract

**Background:** Bullying tends to peak during adolescence, and it is an important risk factor of self-harm and suicide. However, research on the specific effect of different sub-types of bullying is limited.

**Objective:** The purpose of this study is to examine the associations between four common forms of bullying (verbal, physical, relational, and cyber) and self-harm, suicidal ideation (SI), and suicide attempts (SA).

**Method:** This was a cross-sectional study of a sample including 4,241 Chinese students (55.8% boys) aged 11 to 18 years. Bullying involvement, self-harm, SI, and SA were measured via The Juvenile Campus Violence Questionnaire (JCVQ). The association was examined through multinomial logistic regression analysis, adjusted for demographic characteristics and psychological distress.

**Results:** Bullying victimization and perpetration were reported by 18.0 and 10.7% of participants. The prevalence of self-harm, SI, and SA were 11.8, 11.8, and 7.1%, respectively. Relational bullying victimization and perpetration were significantly associated with SI only, SI plus self-harm, and SA. Physical bullying victimization and perpetration were risk factors of self-harm only and SA. Verbal victimization was significantly associated with SI only. Cyber perpetration was a risk factor of SA.

**Conclusions:** The findings highlight the different effects of sub-types of bullying on self-harm and suicidal risk. Anti-bullying intervention and suicide prevention efforts should be prior to adolescents who are involved in physical and relational bullying.

## Introduction

Suicide is a substantial public health concern worldwide and is the third leading cause of death among youth aged 15–19 (1). In fact, more than 79% of global suicides occurred in low- and middle-income countries (1). In China, close to 2 million people attempt suicide and about 12.5% of them complete suicide every year (2). Furthermore, suicide has become the leading cause of death among Chinese young adults (2). Evidence significantly demonstrates that the presence of suicidal ideation (SI, thoughts and plans of ending one's life) and suicide attempts (SA, engagement in potentially self-injurious behavior that does not result in death) are the most important risk factors for suicide (3). According to a population-based study, the prevalence of SI and SA among Chinese adolescents was ~23 and 4%, respectively (4). In response to the high prevalence, researchers have identified risk factors for SI and SA, which range from psychopathology to interpersonal adversity, such as bullying (5).

Bullying is defined as intentional, repeated, and harmful aggressive behavior with an imbalance of power between the perpetrators and the victims. Bullying behavior can occur in a range of contexts including schools, communities, and through electronic means (6, 7). Bullying victimization and perpetration have been conceptualized into three common sub-types, including physical (e.g., hitting, kicking, chasing), verbal (e.g., teasing, name-calling), and social or relational (e.g., excluding or ostracizing from social situations, spreading rumors) (8, 9). In addition, the rapid development and widespread application of online communication have led to the emergence of cyber bullying, which is described as electronic aggression with harmful words or photographs through the computer or cell phone (10). The prevalence of bullying tends to peak during adolescence (11). Over one third of adolescents have experienced traditional bullying (e.g., verbal, physical, and relational) worldwide, whereas more than half of adolescents have reported cyber bullying (12, 13). Some previous research has also indicated that youth rarely experience cyber bullying independent of traditional bullying (14). Therefore, cyber bullying should be included when investigating different sub-types of bullying behavior in addition to verbal, physical, and relational bullying (15, 16).

Empirical evidence suggests that bullying is significantly associated with mental health problems (17), such as anxiety, depression, and psychosomatic symptoms (18, 19). In addition, adolescents who have been bullied are at a greater risk for self-harm and suicidal behavior than those who have not been a victim of bullying (20, 21). Critically, self-harm often co-occurs with SI and SA (22). However, few studies have explored the relationship of bullying perpetration, self-harm, and suicide risks, especially in eastern countries (23, 24). Klomek et al. found that bullying perpetration can predict subsequent SI and SA above and beyond other risk factors such as substance use and functional impairment (25). Therefore, besides victimization, perpetration must be incorporated into the analysis when examining associations between bullying, self-harm, and suicidal behaviors (26).

Despite the underestimation of bullying perpetration, the effect of specific sub-types of bullying behavior is poorly understood (27, 28). Although these sub-types are highly related to each other, they may be associated with adverse health outcomes in different patterns (29). For instance, Espelage and Holt found that youth who engaged in physical bullying had comparatively higher rates of self-harm, SI, and SA than those who were involved in verbal bullying (30). Arango et al. found that all sub-types of bullying victimization and perpetration, except for physical perpetration, were associated with an increased risk of SI. In addition, all forms of bullying, except for relational perpetration, were significantly associated with increased risk of SA (8). These studies claim that different sub-types of bullying behavior may have unique effects on self-harm and suicidal risk. However, these findings are controversial and based on a small adolescent sample (8). Therefore, it is necessary to examine the specific associations between different sub-types of bullying and self-harm, SI, and SA in a large and representative sample.

Furthermore, less is known about the adverse health-related outcomes of cyber bullying (31). Williams et al. found that cyber bullying could be a better predictor of depressive symptoms, SI, and SA as compared to verbal, physical, and relational bullying (11). Therefore, it is interesting to explore which one is the strongest risk factor of self-harm, SI, and SA in the full range of bullying victimization and perpetration, including verbal, physical, relational, and cyber (32).

Taken together, few studies have examined unique associations between bullying behavior and self-harm, SI, and SA in context of the four common sub-types of victimization and perpetration (verbal, physical, relational, and cyber). However, exploring the relationship between the severity of different sub-types of bullying and suicidal risk is particularly important for efficient prevention. More specifically, better understanding the effect of different sub-types of bullying could help medical providers to identify adolescents at the highest risk for suicidal behavior (33). Nevertheless, most of the previous studies on this issue were conducted in western countries and/or in a small sample (8, 11). Hence, it is necessary to extend the existing literature based on a large sample of adolescents in eastern and developing countries, such as China.

In order to address these gaps, the goal of the current study is to identify specific associations between sub-types of bullying and self-harm, SI, and SA in a large and random sample from a Chinese adolescent population. We aim to answer two main questions in the study: first, whether the four sub-types (verbal, physical, relational, and cyber) of bullying victimization and perpetration have distinct effects on self-harm, SI, and SA; and second, which sub-type of bullying has the strongest effect after adjusting for demographic characteristics and psychological distress.

## Materials and Methods

### Procedures

This study was a cross-sectional survey, conducted from March to October 2017. The participants were recruited via cluster sampling in Hubei Province, which is located in central China. First, we selected two cities (E'zhou and Xiaogan) randomly in the province. Second, with the help of local educational bureaus, we sampled three junior high schools and three senior high schools in each chosen city. Then, we selected two or three classes from 7th to 12th grade in every chosen school. Finally, all students from the chosen class were invited to the study as participants. All participants were required to complete the paper questionnaire independently, with the mean completion time between 20 to 30 min.

All students and their parents or guardians who participated in the study voluntarily signed informed written consent before investigation. The purpose of the study and the questionnaire sections were explained to them by investigators. The students were assured of the anonymity and confidentiality of the information provided in the self-reported questionnaires. The study received the approval from the sample schools and the Medical Ethics Committee of Tongji Medical College, Huazhong University of Science and Technology. More information about the study has been described in https://osf.io/gckvu/?view_only=16e86f59733f45459c8de58fb1777046.

### Participants

Questionnaires were sent out to 4,500 participants. After field investigation, we excluded 168 questionnaires due to some students having invalid responses (missing items of whole questionnaire were more than 15%). Then, based on the aims of this study, we excluded 91 questionnaires since participants did not provide information about the key variables of interest (e.g., bullying, self-harm, suicidal ideation, or suicide attempts). Finally, 4,241 (94.2%) questionnaires were included in the statistical analysis.

### Bullying

The Juvenile Campus Violence Questionnaire (JCVQ) was developed by Chinese scholars to survey aggressive and violent behaviors on campus and had good validity and reliability (Cronbach's alpha was 0.91) in Chinese adolescent populations (34). The JCVQ provides a broader coverage of juvenile violent behaviors and assesses 36 items referring to victimization or perpetration covering 9 dimensions of interest: physical aggression, self-harm, suicide, sexual abuse, verbal aggression, relational aggression, cyber violence, tools violence (aggression with weapon), and peer pressure. All 36 items were assessed with the same question, asking how often the event occurred during the past year. Responses were scored on a 4-point, Likert-type scale, where 1 = “never,” 2 = “sometimes,” 3 = “often,” and 4 = “almost.” The internal consistency reliability (Cronbach's alpha) of the JCVQ in the study was 0.90. The Cronbach's alpha score for 9 dimensions of the JCVQ ranged from 0.83 to 0.94.

For this study, four sub-types of bullying behavior were measured via 16 items (8 items for victimization and 8 items for perpetration) from four dimensions (verbal aggression, physical aggression, relational aggression, cyber violence) using the JCVQ. Specifically, (1) verbal victimization: “I was called nasty name.” “I was made fun of.” (2) Physical victimization: “I was hit, kicked, pushed, or shoved.” “My belongings were taken or damaged.” (3) Relational victimization: “I was excluded from the group or completely ignored.” “Someone told lies or spread rumors about me and/or tried to make others dislike me.” (4) Cyber victimization: “I was called nasty name or made fun of online.” “Someone spread rumors about me online.” According to the well-accepted definition that bullying refers to some repetitive aggressive behaviors (35), participants were considered to be involved in a form of bullying victimization (coded 1) if the response of any specific item was 3 = “often” or 4 = “almost,” whereas they were coded 0 if the response was 1 = “never” or 2 = “sometimes.” Then, bullying perpetration was measured in the same pattern mentioned above.

The JCVQ does not require respondents to define themselves as bullies or victims, but rather asks about the frequency of each event related to bullying behavior. The instructions for the JCVQ are straightforward but do not provide a definition of bullying. This is because prior research has demonstrated that even when people do not label themselves as victims or bullies, they still suffer negative effects (36, 37).

In bullying involvement, “victim only” was defined as participants involved in any sub-type of bullying victimization but not engaging in perpetration. “Bully only” was classified as youth who perpetrated bullying behavior to others but were not bullied. “Bully-victim” was defined as a youth who experienced both bullying victimization and perpetration. Those who neither bullied others nor were bullied by others were classified as “non-involved” (38, 39).

### Self-Harm, Suicidal Ideation, and Suicide Attempts

Self-harm, suicidal ideation (SI), and suicide attempts (SA) were measured through three items from JCVQ. (1) Self-harm: “I hurt myself intentionally by cutting or burning my skin.” (2) SI: “I thought about killing myself.” (3) SA: “I try to commit suicide.” Participants were considered to have self-harm, SI, or SA (coded 1) if the response was 2 = “sometimes,” 3 = “often,” or 4 = “almost,” while they were coded 0 if the response was 1 = “never” (40).

### Psychological Distress

The 10-item Kessler Psychological Distress Scale (K-10) was used to measure symptoms of psychological distress occurring over the last 4 weeks (41). The K-10 was most often treated as a unidimensional scale and has good validity in community and clinical settings among adolescent and adult populations (42). The Chinese version of K-10 has good validity and reliability (Cronbach's alpha was 0.80) among the Chinese population according to previous findings (43). Each item was scored on a 5-point Likert scale where 1 = “none of the time,” 2 = “a little of the time,” 3 = “some of the time,” 4 = “most of the time,” and 5 = “all of the time.” Responses were summed to generate a total score ranging from 10 to 50, with higher scores indicating greater psychological distress (44). Multiple cut-offs were used to split populations into four groups representing low (10–15 score), moderate (16–21 score), high (22–29 score), and very high (30–50 score) levels of psychological distress (41). The Cronbach's alpha of the K-10 in this study was 0.89.

### Demographic Variables

Demographic variables included gender, grade (from 7th to 12th), family composition (participant lives in a family with: 1= two biological parents, 2 = a single biological parent, 3 = others) (45), caregiver (1= parents, 2 = grandparents, 3 = other), caregiver's education (1 = primary school or less, 2 = junior high school, 3 = senior high school, 4 = college or more), and family income (average family income per month in RMB: 1 = ~ 999, 2 = 1000 ~ 2999, 3 = 3000 ~ 4999, 4 = 5000 ~ 7999, 5 = 8000 ~).

### Statistical Analysis

First, demographic characteristics of participants and prevalence of bullying, self-harm, SI, and SA were summarized by descriptive statistics [n (%)]. Second, the chi-square test was used to compare the prevalence of self-harm, SI, and SA in different sub-types (verbal, physical, relational, and cyber) of bullying. Pearson's correlation was used among four sub-types of bullying, self-harm, SI, and SA.

Then, in order to examine the associations between sub-types of bullying and self-harm, SI, and SA, two models of multinomial logistic regression analyses were performed separately. In model 1, we included four sub-types of bullying victimization and perpetration (1= yes, 0 = no) as independent variables. In model 2, in addition to the four sub-types of bullying victimization and perpetration, we included gender, grade, family composition, caregiver, caregiver's education, family income, and psychological distress score as confounding variables. As some of the participants would have simultaneously experienced self-harm, SI, and SA, we classified participants into five categories: 0 = none (without self-harm, SI, and SA), 1= self-harm only, 2 = SI only, 3 = SI plus self-harm (simultaneous SI and self-harm but not SA), and 4 = SA (regardless of whether they experienced SI or self-harm) (4). The dependent variable of the multinomial logistic regression analysis was the five categories (0, 1, 2, 3, and 4).

The associations were reported via odd ratios (OR) and 95% confidence intervals (95% CI). The significance level was set at *p* < 0.05. All data was analyzed by SPSS 23.0.

## Results

### Demographic Characteristics of the Participants

Among 4,241 participants, 2,306 were boys (55.8%), 1,828 were girls (44.2%), and 107 were missing. Their ages ranged from 11 to 18 years. The average age was 14.36 ± 1.80. There were slightly more junior high school students (grades 7 to 9) than senior high school students (grades 10 to 12) (53.6 vs. 46.4%). Most participants lived in a two biological parent family (89.3%), while 8.2% were from a single biological parent family and 2.5% from other type of family. The distribution of caregiver, caregiver's education, and family income is shown in Table 1.

**Table 1:** Demographic characteristics and bullying involvement of participants.

**Variables**	**N**	**%**
**Gender^a^**		
Boy	2,306	55.8
Girl	1,828	44.2
**Grade**		
7th	783	18.5
8th	759	17.9
9th	730	17.2
10th	710	16.7
11th	733	17.3
12th	526	12.4
**Family composition^a^**		
Two biological parents	3,733	89.3
Single biological parent	343	8.2
Others	106	2.5
**Caregiver^a^**		
Parents	3,702	88.0
Grandparents	413	9.8
Other	92	2.2
**Caregiver's education^a^**		
Primary school or less	452	11.0
Junior high school	1,857	45.2
Senior high school	1,380	33.6
College or more	418	10.2
**Family income (RMB)^a^**		
~ 999	202	5.1
1000 ~ 2999	1,140	29.0
3000 ~ 4999	1,698	43.2
5000 ~ 7999	656	16.7
8000 ~	236	6.0
**Psychological distress^a^**		
Low	885	21.8
Moderate	1614	39.8
High	1067	26.3
Very high	487	12.0
**Bullying involvement**		
Not-involved	3,413	80.5
Victim only	376	8.9
Bully only	66	1.6
Bully-victim	386	9.1
Total	4,241	100.0

a *There was missing data (gender = 107, family composition = 59, caregiver = 34, caregiver's education = 134, family income = 309, psychological distress = 188)*.

### Prevalence of Bullying, Self-Harm, Suicidal Ideation, and Suicide Attempts

In the study, 19.5% (828) of participants were involved in bullying behavior during the last year. With respect to bullying status, 8.9% (376) of participants were victim only, 1.6% (66) were bully only, and 9.1% (386) were bully-victim (Table 1). The mean and standard deviations (SD) for the total psychological distress score was 20.93 ± 6.98.

Prevalence of self-harm, suicidal ideation (SI), and suicide attempts (SA) were 11.8% (502), 11.8% (500), and 7.1% (300), respectively. Of the participants, 18.0% (762) reported at least one subtype of bullying victimization in the last year. The prevalence of the four sub-types of bullying victimization were 11.9% (verbal), 10.6% (physical), 4.0% (relational), and 4.8% (cyber). In bullying perpetration, 10.7% (457) of adolescents bullied others with any sub-type of bulling behavior. The prevalence of the four sub-types of bullying perpetration were 7.9% (verbal), 5.3% (physical), 4.2% (relational), and 3.6% (cyber). In the chi-square tests, adolescents involved in any form of bullying victimization or perpetration had higher rates of self-harm, SI, and SA than those who were not engaged in the sub-type of bullying (*p* < 0.001) (Table 2).

**Table 2:** Prevalence of self-harm, suicidal ideation, and suicide attempts by sub-types of bullying [*n* (%)].

**Sub-types of bullying**	**Total (*n* = 4,241)**	**Self-harm** **(*n* = 502)**	**Suicidal ideation** **(*n* = 500)**	**Suicide attempts** **(*n* = 300)**
**Verbal victimization**				
Yes	505 (11.9)	177 (35.0)^***^	195 (38.6)^***^	142 (28.1)^***^
No	3736 (88.1)	325 (8.7)	305 (8.2)	158 (4.2)
**Physical victimization**				
Yes	449 (10.6)	184 (41.0)^***^	188 (41.9)^***^	150 (33.4)^***^
No	3792 (89.4)	318 (8.4)	312 (8.2)	150 (4.0)
**Relational victimization**				
Yes	171 (4.0)	120 (70.2)^***^	137 (80.1)^***^	121 (70.8)^***^
No	4070 (96.0)	382 (9.4)	363 (8.9)	179 (4.4)
**Cyber victimization**				
Yes	203 (4.8)	121 (59.6)^***^	123 (60.6)^***^	112 (55.2)^***^
No	4038 (95.2)	381 (9.4)	377 (9.3)	188 (4.7)
**Verbal perpetration**				
Yes	336 (7.9)	146 (43.5)^***^	160 (47.6)^***^	133 (39.6)^***^
No	3905 (92.1)	356 (9.1)	340 (8.7)	167 (4.3)
**Physical perpetration**				
Yes	224 (5.3)	135 (60.3)^***^	136 (60.7)^***^	114 (50.9)^***^
No	4017 (94.7)	367 (9.1)	364 (9.1)	186 (4.6)
**Relational perpetration**				
Yes	180 (4.2)	121 (67.2)^***^	135 (75.0)^***^	116 (64.4)^***^
No	4061 (95.8)	381 (9.4)	365 (9.0)	184 (4.5)
**Cyber perpetration**				
Yes	152 (3.6)	117 (77.0)^***^	127 (83.6)^***^	119 (78.3)^***^
No	4089 (96.4)	385 (9.4)	373 (9.1)	181 (4.4)

*** *p < 0.001*.

### Associations Between Sub-types of Bullying and Self-Harm, Suicidal Ideation, and Suicide Attempts

Pearson's correlations among sub-types of bullying, self-harm, SI, and SA were displayed in Table 3. In Collinearity diagnosis of logistic regression analysis, Eigenvalue ranged from 0.226 to 4.847, Condition Index ranged from 1.000 to 4.631, and Variance Proportions ranged from 0.01 to 0.56. The results indicated that four sub-types of bullying victimization and perpetration were independently associated with self-harm, SI, and SA.

**Table 3:** Correlations among sub-types of bullying, self-harm, suicidal ideation, and suicide attempts.

**Variables**	**1**	**2**	**3**	**4**	**5**	**6**	**7**	**8**	**9**	**10**
1. Verbal victimization	-									
2. Physical victimization	0.42^***^	-								
3. Relational victimization	0.43^***^	0.44^***^	-							
4. Cyber victimization	0.49^***^	0.40^***^	0.55^***^	-						
5. Verbal perpetration	0.65^***^	0.39^***^	0.49^***^	0.43^***^	-					
6. Physical perpetration	0.37^***^	0.49^***^	0.50^***^	0.43^***^	0.51^***^	-				
7. Relational perpetration	0.43^***^	0.38^***^	0.61^***^	0.53^***^	0.54^***^	0.52^***^	-			
8. Cyber perpetration	0.43^***^	0.43^***^	0.66^***^	0.63^***^	0.57^***^	0.57^***^	0.69^***^	-		
9. Self-harm	0.26^***^	0.31^***^	0.37^***^	0.33^***^	0.29^***^	0.35^***^	0.36^***^	0.39^***^	-	
10. Suicidal ideation	0.31^***^	0.32^***^	0.43^***^	0.34^***^	0.33^***^	0.36^***^	0.41^***^	0.43^***^	0.55^***^	-
11. Suicide attempts	0.30^***^	0.35^***^	0.51^***^	0.42^***^	0.37^***^	0.40^***^	0.47^***^	0.54^***^	0.55^***^	0.76^***^

*** *p < 0.001*.

In model 1, without controlling for confounding variables, physical victimization and perpetration were significantly associated with self-harm only. Relational victimization and perpetration, as well as verbal victimization, were significantly associated with SI only. There were significant associations between SI plus self-harm and verbal, physical, and relational victimization as well as physical perpetration. All sub-types of bullying, except for verbal victimization and perpetration, were significantly associated with an increased risk of SA (Table 4).

**Table 4:** Multinomial logistic regression of self-harm, suicidal ideation, and suicide attempts [OR (95% CI)]^a^.

**Variables**	**Self-harm only** **(*n* = 199)**	**Suicidal ideation only (*n* = 124)**	**Suicidal ideation plus self-harm (*n* = 76)**	**Suicide attempts** **(*n* = 300)**
**Model 1**				
Verbal victimization (ref. = no)	1.21 (0.68, 2.11)	4.04 (2.34, 6.98)^***^	2.21 (1.05, 4.65)^*^	1.06 (0.63, 1.79)
Physical victimization (ref. = no)	2.54 (1.64, 3.91)^***^	1.26 (0.69, 2.31)	2.34 (1.21, 4.52)^*^	2.91 (1.93, 4.39)^***^
Relational victimization (ref. = no)	0.91 (0.29, 2.88)	3.18 (1.22, 8.30)^*^	3.32 (1.20, 9.22)^*^	6.97 (3.81, 12.75)^***^
Cyber victimization (ref. = no)	1.66 (0.77, 3.57)	0.34 (0.10, 1.21)	0.97 (0.34, 2.73)	1.91 (1.03, 3.54)^*^
Verbal perpetration (ref. = no)	1.20 (0.61, 2.39)	0.55 (0.25, 1.20)	0.61 (0.23, 1.62)	1.16 (0.63, 2.15)
Physical perpetration (ref. = no)	2.39 (1.26, 4.53)^**^	1.97 (0.84, 4.64)	3.89 (1.70, 8.92)^**^	2.32 (1.32, 4.09)^**^
Relational perpetration (ref. = no)	1.78 (0.70, 4.50)	5.02 (2.16, 11.69)^***^	2.89 (0.99, 8.41)	3.65 (1.92, 6.94)^***^
Cyber perpetration (ref. = no)	0.49 (0.12, 2.02)	0.23 (0.03, 2.08)	1.59 (0.43, 5.88)	4.23 (1.96, 9.15)^***^
**Model 2**^b^				
Gender (ref. = boy)	1.68 (1.20, 2.35)^**^	0.97 (0.65, 1.47)	3.18 (1.80, 5.62)^***^	1.60 (1.12, 2.29)^*^
Psychological distress score	1.09 (1.06, 1.11)^***^	1.09 (1.06, 1.11)^***^	1.15 (1.11, 1.18)^***^	1.11 (1.08, 1.13)^***^
Verbal victimization (ref. = no)	1.03 (0.54, 1.96)	3.60 (1.96, 6.62)^***^	2.01 (0.85, 4.74)	0.76 (0.40, 1.43)
Physical victimization (ref. = no)	2.43 (1.49, 3.96)^***^	1.04 (0.53, 2.01)	2.08 (0.98, 4.42)	2.86 (1.77, 4.63)^***^
Relational victimization (ref. = no)	1.16 (0.35, 3.83)	3.76 (1.31, 10.76)^*^	3.92 (1.25, 12.29)^*^	10.90 (5.43, 21.87)^***^
Cyber victimization (ref. = no)	1.80 (0.79, 4.12)	0.34 (0.11, 1.27)	0.70 (0.21, 2.36)	1.76 (0.84, 3.70)
Verbal perpetration (ref. = no)	1.52 (0.72, 3.21)	0.51 (0.22, 1.21)	0.83 (0.28, 2.45)	1.43 (0.70, 2.96)
Physical perpetration (ref. = no)	2.31 (1.13, 4.73)^*^	1.83 (0.69, 4.86)	4.70 (1.86, 11.86)^**^	2.79 (1.48, 5.28)^**^
Relational perpetration (ref. = no)	2.06 (0.78, 5.46)	3.68 (1.37, 9.85)^*^	3.58 (1.09, 11.72)^*^	3.19 (1.48, 6.89)^**^
Cyber perpetration (ref. = no)	0.51 (0.12, 2.27)	0.45 (0.05, 4.39)	1.79 (0.39, 8.31)	4.52 (1.77, 11.56)^**^

a *The reference category for the dependent variables were none (without self-harm, suicidal ideation, and suicide attempts)*.

b *Not significant confounding variables: grade, family composition, caregiver, caregiver's education, and family income*.

*** *p < 0.001*,

** *p < 0.01*,

* *p < 0.05*.

In model 2, after controlling for confounding variables, results were similar to that of model 1 for self-harm only and SI only. SI plus self-harm was significantly associated with relational victimization and perpetration as well as physical perpetration. All sub-types of bullying, except for verbal victimization and perpetration as well as cyber victimization, were significantly associated with increased risk of SA (Table 4).

Additionally, the results showed that the psychological distress score was significantly associated with self-harm, SI, and SA. Compared with boys, girls had a greater risk of experiencing self-harm only, SI plus self-harm, and SA. Grade, family composition, caregiver, caregiver's education, and family income had no significant association with the dependent variable.

## Discussion

This is the first study to examine the effects of different sub-types (verbal, physical, relational, and cyber) of bullying victimization and perpetration on self-harm, suicidal ideation (SI), and suicide attempts (SA) through a large and random sample of adolescents in an Eastern country. First, we found that not all forms of bullying were significantly associated with self-harm, SI, and SA after controlling for some confounding variables, such as psychological distress. Most important, physical and relational bullying, in terms of both victimization and perpetration, might be the stronger risk factors for self-harm and suicide than verbal and cyber bullying. These findings contribute new information concerning the association between bullying and suicidal behavior among adolescents. Researchers could benefit from a better understanding of the specific effect of different sub-types of bullying on suicide.

As we expected, the effect of different sub-types of bullying victimization and perpetration on elevated risk of self-harm, SI, and SA were unique. First, physical bullying was positively associated with self-harm only, SI plus self-harm, and SA, while verbal victimization was associated with SI only. The finding is consistent with previous work, which indicated that physical bullying has a more serious impact on suicidal thoughts and behaviors than verbal bullying among youth (30). On the one hand, the different impact of these two forms of bullying may be rooted in that verbal bullying is more common than physical bullying among adolescents, which affects the risk to a lesser degree (46, 47). On the other hand, the involvement of physical bullying could put adolescents in situations where they are actually injured with physical pain or a threat of injury. Exposure to painful and provocative events could make adolescents more likely to engage in behavior leading to suicide (48).

Second, our results revealed that relational bullying was a strong risk factor for SI and SA, though the association between relational bullying and self-harm only was not significant. A previous study found that relational bullying (social exclusion and rumor spreading) had the strongest association with mental health problems, independent of verbal and physical bullying (49). Another study suggested that relational bullying may be especially detrimental to adolescent adjustment (50). This form of bullying generally causes a more adverse impact on adolescent self-esteem and social status than other forms of bullying (33). Our study extends these findings, highlighting that relational bullying has a stronger association with suicidal risk, independent of other forms of bullying behavior (51).

Relational bullying behavior, such as social exclusion from a group, is subtle and difficult to detect. Therefore, it is less likely to get appropriate attention from adults. This may contribute to the reason why the behavior persists for a longer time and makes self-defense more difficult, which further lead to stress and isolation (52). Adolescents may be particularly sensitive to social exclusion and rumor spreading as it deprives them of their social networks. During adolescence, acceptance and popularity within peer group are critical since youth individuate from their parents (53). Moreover, in this period, adolescents' social-cognitive skills develop rapidly. Therefore, relational bullying may have a more severe impact on adolescents' mental health due to the increased salience of peer relationships and sensitivity to peer rejection during this developmental period (46).

Most researchers treat verbal, physical, and relational bullying as one type called traditional bullying or school bullying (4, 16). It is hard to find specific characteristics of sub-types of bullying behavior and underlying distinct effects on adverse physical and/or mental health consequences. According to the results from the current study, severity of verbal, physical, and relational bullying victimization and perpetration for self-harm, SI, or SA are different. Therefore, it is more suitable to treat different forms of bullying behavior as independent variables when exploring the relationship between bullying and subsequent health problems.

Moreover, our results indicate the unique contribution of cyber bullying in suicide risk. Specifically, only cyber perpetration was significantly associated with SA. This finding was not in line with previous studies, which indicated that cyber bullying could have a more harmful effect on suicide than traditional bullying (21). The discordance may stem from the different classification of bullying behavior. Prior work generally did not distinguish verbal, physical, and relational bullying as a certain sub-type of bullying to compare with cyber bullying. It would weaken the effect of a specific form of bullying on mental health outcomes. Although cyber victimization was not a risk factor of self-harm and suicide risk, it could leave youth feeling extremely isolated and/or helpless, because cyber bullying is not restricted to school campuses and can happen at any time (54).

The prevalence of bullying in this study was lower than that reported in other studies (12, 55). First, the difference in prevalence may result from variations of cultural and economic backgrounds of different countries or regions (47). Second, it could stem from different measurements and cut-off values of bullying behavior in various studies (12). For instance, in the current study, we took a more stringent cut-off value and participants were classified as a victim or perpetrator if any bullying behavior “often” or “almost” happened, while adolescents were identified to have experienced bullying when the frequency was “sometimes” in a recent study (4). In addition, the prevalence of cyber bullying was also lower than reported in other studies, which were mainly conducted in Western developed countries (56, 57). One possible explanation is that most of the junior and senior high students in China attend boarding school. Students stay at school for five or six days a week and they are not allowed to use mobile phones or other online devices at school.

Over and above different sub-types of bullying, we found that psychological distress is significantly associated with self-harm, SI, and SA. Existing literature has demonstrated that there is a positive correlation between bullying experiences and psychological distress (58). Previous researchers have indicated that severe psychological distress is a major risk factor for suicidal behavior (59). Hence, we included psychological distress as a confounder when we examined the relationship between bullying and suicide. The finding may be beneficial for better understanding of predictive factors for suicide risk.

## Limitations

There are several limitations. First, the cross-sectional design and self-reported data limit our study to draw causal associations between bullying and self-harm, SI, and SA. Future studies could benefit from the use of a longitudinal, multi-informant, multi-method design. Second, the current study dichotomized each of the sub-types of bullying as independent variables and did not consider the co-occurrence of different forms of bullying. It would be beneficial to explore the specific effect on self-harm and suicidal risk, but the cumulative effect of bullying cannot be examined. Further, we did not consider other possible confounding variables, such as school environment, sexual orientation, or obesity, which may moderate the association between bullying and suicidality (60, 61). Future research should include more potential cofounders. Finally, although the sample size was large, the study was conducted within one province of China. The extent to which this sample represents is unclear. Future research can recruit more participants in several representative provinces in China via a multi-center sampling design.

## Implications

The findings provide valuable implications for prevention strategies to decrease rates of bullying and suicide. Results from the current study indicated that relational bullying could be a strong risk factor for suicide in all sub-types of bullying. This finding supports the role of thwarted belongingness in predicting suicide risk, which is an essential component of the Interpersonal Theory of Suicidal Behavior (62). Adolescents bullied in a relational way may suffer more unbearable mental pain and lack of belonging, which could increase the risk of suicidality (63). Therefore, it is important to reinforce interpersonal connectedness in youth who are victims of relational bullying. Interpersonal connectedness could be improved via participating in group projects, engaging in team activities, or being involved in school events (8). In addition, our results demonstrate that not all sub-types of bullying are significantly associated with self-harm or suicide. This finding supports the importance of differentiating sub-types of bullying behavior, which can help suicide prevention strategies on specific needs for adolescents involving in bullying. On the other hand, researchers are supposed to design more work to delineate how and why different sub-types of bullying victimization and perpetration have distinct associations with physical and psychological health problems among adolescents.

## Conclusions

The findings highlight the specific effects of different sub-types of bullying victimization and perpetration on self-harm, suicidal ideation, and suicide attempts. Different strategies, based on unique characteristics of different forms of bullying behavior, can be more effective than a one-size-fits-all approach in the development of suicide prevention programs. Anti-bullying intervention and suicide prevention efforts should be aimed to adolescents who are involved in physical and relational bullying, as they face a greater risk of self-harm and suicidality.

## Data Availability Statement

The raw data supporting the conclusions of this article will be made available by the authors, without undue reservation.

## Ethics Statement

The studies involving human participants were reviewed and approved by the Medical Ethics Committee of Tongji Medical College, Huazhong University of Science and Technology. Written informed consent to participate in this study was provided by the participants' legal guardian/next of kin.

## Author Contributions

CP and WH as the first authors, developed the initial manuscript and contributed equally to this paper work. SY, JX, and CK were responsible for the data collection and the data analysis. MW, FR, and YH contributed substantially to the revision and refinement of the final manuscript. YY guided the overall design of the study. All authors have read and approved the final manuscript.

## Conflict of Interest

The authors declare that the research was conducted in the absence of any commercial or financial relationships that could be construed as a potential conflict of interest.
